# Endoscopic endonasal approach for clipping of a PICA aneurysm

**DOI:** 10.3171/2020.4.FocusVid.19901

**Published:** 2020-04-01

**Authors:** Ezequiel Goldschmidt, Philippe Lavigne, Carl Snyderman, Paul A. Gardner

**Affiliations:** Departments of 1 Neurosurgery and; 2 Otolaryngology, University of Pittsburgh Medical Center, Pittsburgh, Pennsylvania

**Keywords:** endonasal endoscopic approach, aneurysm clipping, PICA aneurysm, surgical video

## Abstract

This video depicts the case of a 59-year-old woman that presented to the emergency department with the worst headache of her life. CT showed subarachnoid hemorrhage and digital subtraction angiogram demonstrated a right-side posterior inferior cerebellar artery (PICA) aneurysm. Given the medial and ventral position of the aneurysm, deep to the lower cranial nerves, which obviated distal control from an open approach, and the absence of an endovascular option able to reliably preserve the PICA, an endonasal approach was offered. A far medial approach was performed, and the aneurysm was successfully clipped. The patient developed a postoperative CSF leak with persistent posthemorrhagic hydrocephalus treated with reexploration and an eventual ventriculoperitoneal shunt. The patient was discharged without neurological deficits.

The video can be found here: https://youtu.be/_9hsM2CaMow.

## Transcript

This is the case of a posteroinferior cerebellar artery aneurysm that was treated endonasally. This is a 59-year-old woman presenting with a subarachnoid hemorrhage. Here we can see on CT and an angiogram shows a very medial and ventral aneurysm. Given this location, we thought an anterior approach would be best.

**0:38** Of course, the procedure starts with a vascularized nasoseptal flap which extends down to the nasal floor.

**0:50** After posterior septectomy a sphenoidotomy is performed. A small, inverted U-shaped rhinopharyngeal or retropharyngeal flap is performed to ensure separation from the defect and the oropharynx.

This small inferior flap also is part of the reconstruction preventing CSF leak down into the nasopharynx. This gave us access all the way down to the foramen magnum, and so the lower clivus and so the foramen magnum and the clivus can be drilled flushed with the periosteal dura.

**1:17** Here we see this extending all the way out on the left side up to the lateral aspect of the foramen magnum, but on the right side we will drill all the way to the jugular tubercle (the so-called far medial approach). To access this, we provided transpterygoid approach, which requires transection of the vidian nerve, drilling of the base of the pterygoid, and then we proceed with drilling of the remainder of the midclivus. The Eustachian tube is disconnected with needle-tip Bovie, and then we drilled out the remainder of the middle pterygoid wedge and a portion of the base of the middle pterygoid.

**1:48** Here we are drilling just medial to foramen lacerum, which can be seen immediately above the drill and then all the way out into the medial jugular tubercle. This gives us access laterally to have proximal control of the vertebral artery proximal to the takeoff of the PICA. Here, further drilling behind foramen lacerum is seen with the drill immediately behind the carotid artery, which is protected with the bone of its canal.

**2:31** The dura is opened after stripping the periosteal layer; this is using a micro through-cut instrument and flap laterally. We can see the subarachnoid hemorrhage and carefully dissect first the distal and then the proximal vertebral artery. Proximal temporary control is ensured, and the further removal of subarachnoid blood is performed. We can see the sixth nerve exiting the brainstem and the vertebra-basilar junction. In dissecting the aneurysm there is some difficulty in visualizing the proximal PICA, but with some effort we are able to see it. This does cause some small amount of bleeding from this very tenuous aneurysm. A single straight clip is placed initially across the approximal neck of the aneurysm. This does result, unfortunately, in further bleeding from the aneurysm. For control we now place a temporary clip proximally on the vertebral artery and then removal of the clip shows that we still have some bleeding from the distal vertebral as expected, and we are able to replace the clip with some control over the aneurysm. The beauty of this approach is that given how ventral and medial the aneurysm is we are able to trap the artery when we deal with a rupture like this. Our initial clip placement is evaluated. We are able to see the lower cranial nerves distal on the other side of the aneurysm, and we can see, unfortunately, a small residual at the takeoff of the PICA.

**3:36** As you can see, this aneurysm sits directly behind the lower cranial nerves and any open approach would lead to significant manipulation of them and is extremely difficult with distal control. To reposition the clip, we place our proximal clip again. Very significant bleeding again from the aneurysm. Attempted replacements are not particularly successful without distal control. We plan now, after replacement of that clip, for a trapping of the aneurysm. Here we trap the vertebral artery temporarily; there is of course some mild burst suppression used during this part, but with these temporary clips in place we have complete control over the vertebral artery, and we are able to very carefully place our permanent clip across the extra nubbin of aneurysm while preserving the PICA. Here the extra blister aneurysm is treated with a small curved clip. This adjacent area of aneurysm is treated while maintaining patency of the PICA, which is confirmed both with Doppler as well as intraoperative indocyanine green angiographic endoscopy that we see here. This confirms patency of the PICA, which is somewhat difficult to visualize. We then test that patency by proximally clipping the vertebral and ensuring that there is backfilling through the vertebral into the PICA.

**4:18** Reconstruction is performed with multilayer collagen and then a large piece of fascial lata, which covers the entire defect all the way down to the nasopharynx in between the level of the carotid artery. This is filled with fat to prevent the development of an encephalocele, and then the nasoseptal flap is placed over the entire defect. Indocyanine green angiography is again performed to confirm vascularity of both the RP flap and the nasoseptal flap. Postoperative angiogram confirms obliteration of the aneurysm and preservation of PICA.

**5:40** Postoperative course was complicated by posthemorrhagic hydrocephalus, which resulted in a CSF leak which required a revision and ultimately a ventriculoperitoneal shunt for treatment of the hydrocephalus.
